# The efficacy and safety of inositol supplementation in preterm infants to prevent retinopathy of prematurity: a systematic review and meta-analysis

**DOI:** 10.1186/s12886-019-1140-z

**Published:** 2019-06-25

**Authors:** Yang Du, Yue He, Yue-lin Wang, Jian-guo Zhou, Chao Chen

**Affiliations:** 1 Department of Neonatology, Children’s Hospital of Fudan University, 399 Wanyuan Road, Minhang District, Shanghai, 201102 China; 2 Department of Ophthalmology, Peking Union Medical College Hospital, Beijing, China

**Keywords:** Retinopathy of prematurity, Inositol supplementation, Mortality, Meta-analysis

## Abstract

**Background:**

Inositol supplementation has been linked to beneficial effects on reducing the incidence of retinopathy of prematurity (ROP); however, it’s controversial. The meta-analysis aimed to check out the efficacy and safety of inositol supplementation in preterm infants for preventing ROP.

**Methods:**

We conducted searches through PubMed, EMBASE, Medline, Cochrane Central Register of Controlled Trials, Cochrane Database of Systematic Reviews, ClinicalTrials.gov website and conference proceedings. Randomized controlled trials comparing inositol supplementation with placebo were included. Two independent reviewers performed screening, review, and extraction. Statistical analysis was performed using R Project.

**Results:**

Six studies (1194 infants) were proved eligible. In comparison with placebo, inositol supplementation revealed no effect on the incidence of severe ROP (relative risk [RR] = 0.49, 95% confidence interval [CI], 0.18–1.32; heterogeneity, *P* = .02; I^2^ = 66%; low quality of evidence [QOE]), mortality (RR = 1.25, 95% CI, 0.82–1.90; heterogeneity, *P* = .07; I^2^ = 51%; low QOE), all stages of ROP (RR = 0.98, 95% CI, 0.87–1.11; heterogeneity, *P* = .41; I^2^ = 0%; moderate QOE) and other adverse events. Sensitivity analysis showed an increased mortality in the inositol group (RR = 1.55, 95% CI, 1.14–2.11; heterogeneity, *P* = .30; I^2^ = 18%) after removing the study Hallman 1986, and meta-regression showed a significant association between publication year and efficacy of inositol compared with placebo (β = 0.1241; 95% CI, 0.0417–0.0026; z = 2.9527; *p* = .0032).

**Conclusions:**

Based on current evidence, inositol supplementation showed no significant effect on preventing severe ROP, and exploratory sensitivity analysis showed a trend toward an increase on mortality.

**Electronic supplementary material:**

The online version of this article (10.1186/s12886-019-1140-z) contains supplementary material, which is available to authorized users.

## Background

Retinopathy of prematurity (ROP) is a disorder of the immature retina in premature infants, which possibly leads to impairment of vision and even blindness [1]. With advances in management, the rising survival rate of premature infants has unexpectedly increased the long-term morbidities of premature infants, equivalently [2–6]. Several recent studies revealed that the incidence of ROP has increased greatly in both developed and developing countries, such as the United States, Sweden, China and India, which means ROP has become a leading cause of childhood blindness [6–9]. Although there has been effective treatments for severe ROP, they are either invasive such as laser treatment or expensive such as anti-VEGF agents [10]. Besides, several studies indicated propranolol might be a potential effective drug for ROP [11–13]. However, the quality of the studies was not high and the safety of propranolol remained to be proved.

Inositol is a naturally occurring six-carbon sugar derivative found in most foods including breast milk. It is an important component of surfactant, and exists intracellularly as phosphoinositide [14]. Inositol supplementation increases the amount of saturated phosphatidylcholine in surfactant in infants. Although the mechanism has not been clear, these findings indicated an important role for inositol to infants.

In 1986 and 1992, Hallman et al. reported two trials in which treatment of infants with respiratory distress syndrome with inositol could improve survival rate and reduced the incidence of ROP [15, 16]. A Cochrane meta-analysis in 2015 concluded that inositol supplementation potentially reduced preterm death, severe ROP, and severe intraventricular hemorrhage (IVH) [17]. Besides that, another meta-analysis also drew the similar conclusion [18]. However, the results of a recently published large multicenter randomized clinical trial (RCT) didn’t support the previous conclusion, which concluded treatment with inositol did not reduce the risk of type 1 ROP or death vs placebo [19]. Given the early termination, the trial was also not formally powered to make a conclusive assessment. Therefore, we performed a systematic review and meta-analysis to determine the efficacy and safety of inositol supplementation in preterm infants for preventing ROP.

## Methods

### Study selection

We included randomized clinical trials that reported the efficacy and safety of applying inositol in the prevention of ROP. Eligible studies should meet the following criteria: (1) Subjects of study were neonates with gestational age less than 32 weeks or birthweight < 2000 g and without ROP before inclusion. (2) Intervention referred to applying inositol by intravenous injection or oral feeding (including milk additive). (3) Eligible studies should have at least one of the following outcomes: mortality, the incidence of severe ROP or any stage of ROP. (4) Only prospective randomized clinical trials were considered to be included. Observational studies and non-randomized clinical trials were considered for exclusion.

### Search strategy

PubMed, Medline, EMBASE, Cochrane Central Register of Controlled Trials, Cochrane Database of Systematic Reviews, and clinicaltrials.gov website was searched from inception to November 1st, 2018 without language restrictions. Relevant conference proceedings were used to identify additional literature.

We used the following search terms: (preterm OR neonates) AND (inositol OR myo-inositol), and the articles relevant with ROP were screened manually. One author executed the search strategy and another author independently peer-reviewed the strategy. An independent librarian peer-reviewed the strategy.

To identify studies and determine eligibility, two authors independently reviewed titles and abstracts for inclusion, and full manuscripts and further relevant references were examined if necessary. If two authors disagree, a third researcher would participate in the decision discussion.

### Data extraction

The name of author, year of publication, study design and outcomes were extracted from each study. The primary outcomes were (1) incidence of severe ROP (defined as incidence of ROP stage ≥3 or level of ROP meeting criteria for surgical intervention) (2) mortality (defined as mortality related to all neonatal conditions during the whole follow-up period). The secondary outcomes were any stage of ROP, BPD, suspected or proven NEC, surgical NEC, all grade of IVH, severe IVH (grade III or IV), neonatal seizures, hearing impairment (hearing test of one ear or both ears failed).

### Risk for Bias

The quality of included RCTs were assessed by using the Cochrane Collaborative’s risk for bias assessment tool [20]. Each evaluator assessed the risk of bias including selection bias (random-sequence generation and allocation implementation), performance bias (blinding of participants and interveners), attrition bias (miss or quit after randomization), detection bias (blinding of outcome evaluations), reporting bias (selective outcome reporting), and other potential bias. Each criterion was assessed by scoring ‘low risk for bias’, ‘high risk for bias’, or ‘uncertain risk for bias’. Any disagreement was solved by discussion.

### Exploration of heterogeneity and statistical analysis

We used the intention-to-treat principle. Statistical analysis was performed using R version 3.5.1 (R Project for Statistical Computing) with R package (meta, metafor, metareg). All outcomes were reported with relative risk (RR) and 95% confidence interval (CI). We assessed heterogeneity among multiple studies by using I^2^ method with the χ^2^ test to calculate *P* value. If the homogeneity test showed *P* > 0.1 and I^2^ < 50% and there is high homogeneity in designing between included studies, we performed fixed effect model (Mantel-Haenszel method) to combine the summary statistics. Since higher I^2^ value indicated high statistical heterogeneity or there was significant high heterogeneity in designing between included studies, we performed random effect model (DerSimonian-Laird method) to combine the summary statistics. Additionally, we did sensitivity meta-analysis and meta-regression for primary outcomes to explore high heterogeneity. We planned to conduct funnel plot analysis and Egger’s test to evaluate the possibility of publication bias if the number of studies in an analysis exceeded 10. The results reported with 95% CI, and the 5% level (*P* < 0.05) was considered to indicate statistical significance.

### Grading the quality of evidence

We used the GRADE (Grading of Recommendations Assessment, Development, and Evaluation) approach to evaluate the quality of evidence [21]. RCTs started with an initial rating of high while nonrandomized comparative studies with low. For each outcome, we assessed method limitations of the included studies, precision, directness, consistency, and the likelihood of publication bias.

## Results

### Description of the evidence

**Fig. 1:**
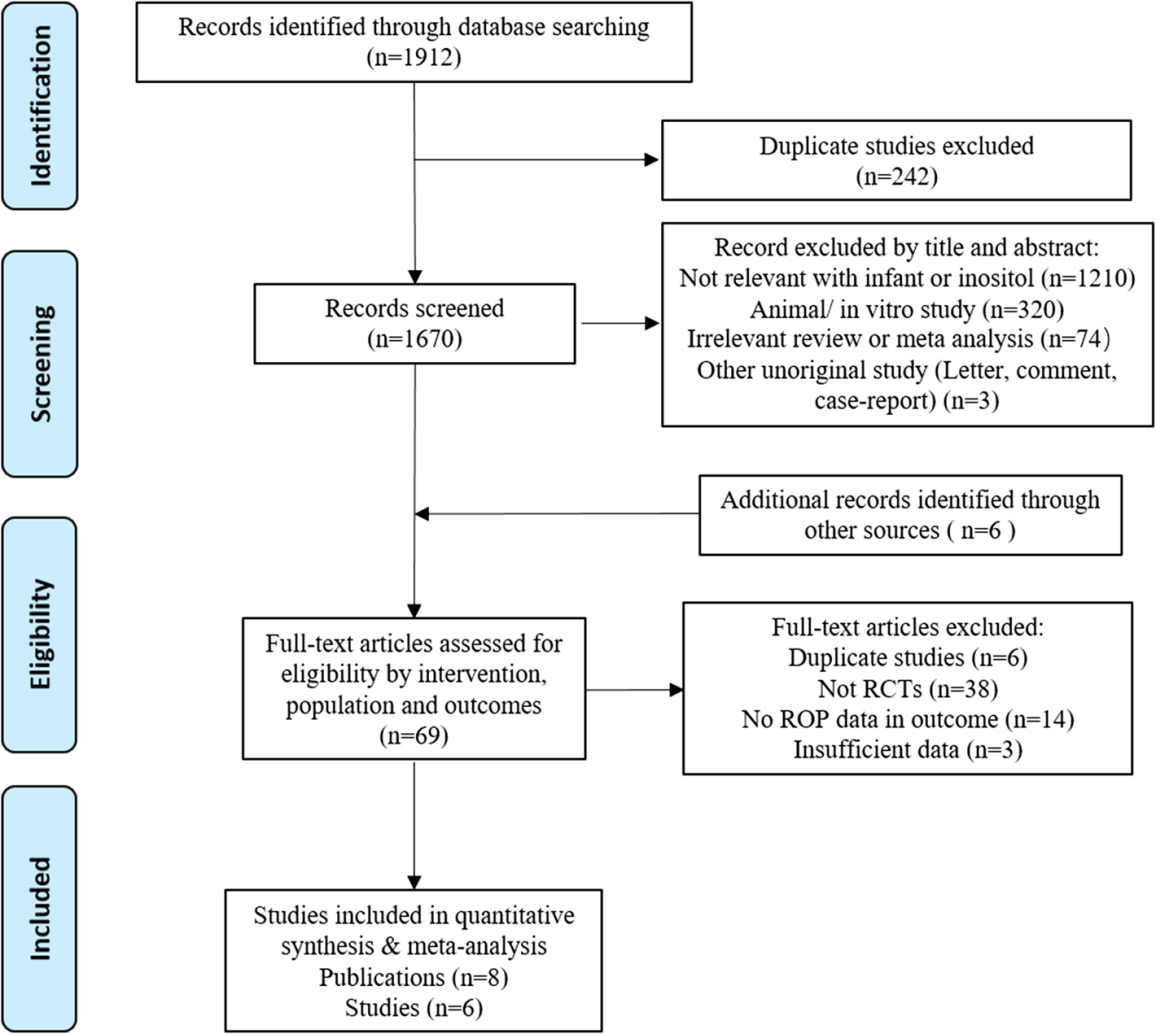
Flow of Study Selection. RCT indicates randomized controlled trial

The online search identified 1670 articles. After excluding 1607 records by screening the titles, abstracts and trial registries, a total of 63 manuscripts were fully examined (Fig. 1). We finally enrolled 6 randomized clinical trials [15, 16, 19, 22–26] for meta-analysis.

These six RCTs involving a total of 1194 preterm infants with BW < 2000 g, of which three [15, 16, 22–24] included 355 preterm infants with respiratory distress syndrome (RDS) and requiring mechanical ventilation, two [25, 26] included 201 preterm infants with 23^0/7^ to 29^6/7^ weeks GA and one [16] included 638 extremely preterm infants born before 28^0/7^ weeks of gestation. The main baseline characteristics selected as gestational age and weight, which were well balanced between the inositol and placebo group of all studies. In addition, none of the infants enrolled had major congenital anomalies, eye anomalies, or moribund conditions. All of the trials were finished in the United States.

All premature infants were assigned to the inositol group or placebo group. Three RCTs [15, 19, 26] supplied inositol or placebo to infants by intravenous injection firstly and then oral feeding with repeat dose (duration from 5 days to 10 weeks). Phelps 2013 [25] injected inositol or placebo intravenously with a single dose in 20 min. Friedman 1995 [23, 24] fed infants with high-inositol formula continuously as intervention and low-inositol formula as placebo control. The trial reported by Hallman in 1986 [15] didn’t use surfactant but all of the other five trials [16, 19, 22–26] reported surfactant administration. All the six RCTs reported number of neonatal or infant deaths, while five trials [16, 19, 23, 25, 26] of which reported number of severe ROP. Number of other complications and adverse events were reported in all or part of these RCTs (Table 1).

**Table 1:** Characteristics of Included Studies

Study	Country	Study Design	Participants	Intervention		Outcomes		Surfactant Administration
				Inositol	Control	Primary	Secondary	
Hallman 1986	US	Single-center randomized double blind placebo-controlled trial	Preterm infants (*n* = 74), BW < 2000 g, with a diagnosis of RDS, requiring mechanical ventilation	IV or po supplemental inositol given daily for ten days	Placebo (5% glucose)	Number of neonatal deaths and infant deaths	Number of infants with BPD, IVH, ROP, NEC, and sepsis	No
Hallman 1992	US	Single-center randomized double blind placebo-controlled trial	Preterm infants (*n* = 233), BW < 2000 g and a PMA of 24.0 to 31.9 weeks at birth, with evidence of RDS, requiring mechanical ventilation.	IV inositol daily for five days, with repeated courses at day 10 and day 20 if necessary (infant continued to require ventilation, required supplemental O_2_ or did not tolerate enteral feeds)	Placebo (5% glucose)	Number of neonatal deaths and BPD	Number of infant death, ROP, IVH (all grades, grade > 2), NEC, and sepsis	Yes^a^
Friedman 1995	US	Double-center randomized placebo-controlled trial	Preterm infants (*n* = 48), BW < 1500 g with a diagnosis of RDS, requiring mechanical ventilation	Feed high-inositol formula (2500 μmol/L inositol) eternally. Duration of supplemental inositol was not reported.	Feed low-inositol formula (242 μmol/L inositol) eternally	Number of infants with ROP	Number of deaths, infants with bacteremia, NEC, IVH (> grade 2), BPD, duration of mechanical ventilation	Yes
Phelps 2013	US	Multi-center randomized double-blind placebo-controlled PK trial	Preterm infants (*n* = 76), with 23^0/7^–29^6/7^ weeks gestation age and ≥ 600 g birthweight, had no major congenital anomalies, and had received no human milk or formula feedings since birth.	IV 5% inositol with a single low (60 mg/kg) (*n* = 25) or high (120 mg/kg) (*n* = 24) dose over 20 min	Placebo (5% glucose)	Pharmacokinetic data for inositol	Number of adverse events in the first 7 days as well as neonatal morbidities from birth through hospital discharge (or 120 days if sooner)	Yes
Phelps 2016	US	Multi-center randomized double-blind phase II clinical trial	Preterm infants (*n* = 125), with 23^0/7^ to 29^6/7^ weeks GA, weighed at least 400 g, and could receive study drug by 72 h after birth, had no major congenital anomalies, severe oliguria, or a moribund state	IV 10, 40 or 80 mg/kg/day inositol (divided every 12 h) from enrolment on day 1 to 3 to 10 weeks of age, to 34 weeks PMA or to discharge. Once feedings were established the same dose of study drug was given eternally.	Placebo (5% glucose)	Population pharmacokinetics data for inositol	Number of type 1 ROP and other adverse events	Yes
Phelps 2018	US	Multi-center randomized double-blind placebo-controlled phase III clinical trial	Extremely preterm infants (*n* = 638) born before 28^0/7^ weeks of gestation, surviving for at least 12 h, admitted to 1 of the 18 Neonatal Research Network centers before 72 h’ postnatal age, without major congenital anomaly, eye anomaly, or moribund condition	IV 40 mg/kg/day inositol (divided every 12 h) from enrolment on day 1 to 3 to 10 weeks of age. Once feedings were established the same dose of study drug was given eternally.	Placebo (5% glucose)	Number of participants with unfavorable outcome, defined as severe retinopathy of prematurity (ROP) or death prior to reaching acute/final ROP status	Number of any type of ROP, type 2 ROP or greater, all-cause mortality, BPD, IVH and other adverse events	Yes

*GA* indicates gestational age; *BW* birthweight, *PMA* postmenstrual age, *IV* Intravenous injection, *po* peros, *ROP* retinopathy of prematurity, *BPD* bronchopulmonary dysplasia, *NEC* necrotizing enterocolitis, *IVH* intraventricular hemorrhage

^a^Part of participants received surfactant for another trial

Among all RCTs, three [15, 16, 23] didn’t clarify the randomization procedure, while the other three [19, 25, 26] specified the use of a computer-based randomization program that allowed complete concealment of the randomization sequence. Four studies [15, 19, 25, 26] masked all the involved physicians, nurses and ophthalmologists. Implementation of masking was not mentioned in two studies [16, 23]. Three trials [19, 25, 26] were registered in a trial registry but the other three [15, 16, 23] were not. Two studies [15, 16] performed interim analysis, which were not registered, and one study [23, 24] had been reported 3 times and the number of neonates enrolled was increasing, which cause high risk of bias. All the RCTs included were assessed by the Cochrane Collaboration’s risk for bias assessment tool [20], and three of which were rated as high quality while others were varying, from moderate to low (Table 2).

**Table 2:** Risk of Bias

Study	Selection Bias		Performance Bias	Detection Bias	Attrition Bias	Reporting Bias	Other
	Random sequence generation	Allocation concealment	Blinding of participants and personnel	Blinding of outcome assessment	Incomplete outcome data addressed	Selective reporting	Other bias
Hallman 1986	Unclear	Low	Low	Low	Low	Unclear	High
Hallman 1992	Unclear	Unclear	Low	Unclear	Low	Unclear	High
Friedman 1995	Unclear	Low	Unclear	Unclear	Low	Unclear	High
Phelps 2013	Low	Low	Low	Low	Low	Low	Low
Phelps 2016	Low	Low	Low	Low	Low	Low	Low
Phelps 2018	Low	Low	Low	Low	Low	Low	Low

### Primary outcomes

#### Severe ROP

**Fig. 2:**
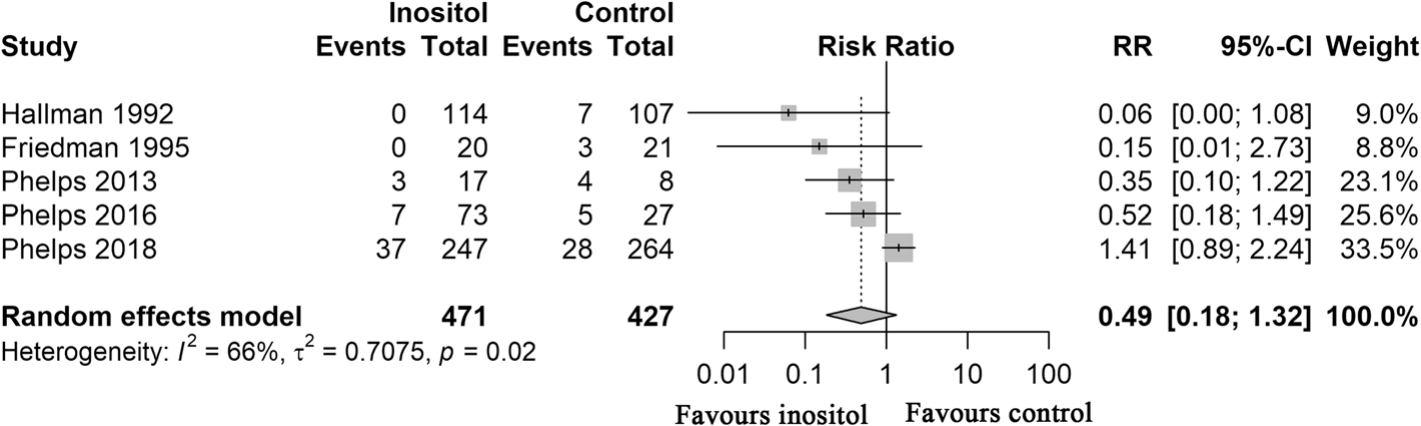
Forest Plot Showing Risk Ratio (RR) in Severe ROP. RR indicates risk ratio; CI, confidence interval; ROP, retinopathy of prematurity Risk ratios were calculated using the DerSimonian-Laird method to combine summary statistics, and data were pooled using a random-effects model.

The primary outcome of this meta-analysis was the incidence of severe ROP. In the inositol group, 47 (9.98%) of the 471 preterm infants from 5 studies [16, 19, 23, 25, 26] developed severe ROP, compared to 47 (11.01%) of 427 in the placebo group. The meta-analysis revealed no statistically significant difference among infants who received inositol supplement versus those who received a placebo (RR = 0.49, D-L random-effects, 95% CI, 0.18–1.32; heterogeneity, *P* = .02; I^2^ = 66%; low QOE; Fig. 2, Table 3).

**Table 3:** GRADE Summary of Findings of Supplemental Inositol Compared to Placebo for Retinopathy of Prematurity

Outcomes	No. of Cases per 1000 Infants		RR (95% CI)	Participants, No.	Studies No.	Quality of Evidence^b^
	Assumed Risk of Placebo^a^	Corresponding Risk of Supplemental Inositol (95% CI)				
Severe ROP	110	54 (19–145)	0.49 (0.18–1.32)	898	5	Low ^c, d^
Mortality	155	194 (127–294)	1.25 (0.82–1.90)	1177	6	Low ^c, e^
All stage of ROP	472	463 (411–524)	0.98 (0.87–1.11)	889	4	Moderate ^e^
BPD	436	414 (314–545)	0.95 (0.72–1.25)	1099	6	Low ^c, e^
Suspected or proven NEC	88	75 (51–109)	0.85 (0.58–1.24)	1189	6	Moderate ^e^
Surgical NEC	37	28 (9–88)	0.76 (0.24–2.38)	834	3	Moderate ^c^
All stage of IVH	392	302 (231–392)	0.77 (0.59–1.00)	429	3	Moderate ^f^
Severe IVH (grade III/IV)	174	118 (78.3–180)	0.68 (0.45–1.03)	1179	6	Low ^c, e^
Late-onset sepsis	194	236 (188–299)	1.22 (0.97–1.54)	1141	5	Low ^c, f^
Seizure	26	27 (11–66)	1.02 (0.41–2.56)	833	3	High
Hearing impairment	107	146 (93–247)	1.36 (0.87–2.31)	605	3	High

^a^The basis for the assumed risk (e.g. the median control group risk across studies) is provided. The corresponding risk (and its 95% CI) is based on the assumed risk in the comparison group and the relative effect of the intervention (and its 95% CI)

^b^The GRADE Working Group grades of evidence are as follows: high quality (further research is very unlikely to change our confidence in the estimate of effect), moderate quality (further research is likely to have an important impact on our confidence in the estimate of effect and may change the estimate), low quality (further research is very likely to have an important impact on our confidence in the estimate of effect and is likely to change the estimate), and very low quality (we are very uncertain about the estimate)

^c^Heterogeneity is highly significant between included trials and hard to be explained

^d^One trial has high risk of bias and one has unclear risk of bias

^e^Two trials have high risk of bias and one has unclear risk of bias

^f^Two trials have high risk of bias

#### Mortality

**Fig. 3:**
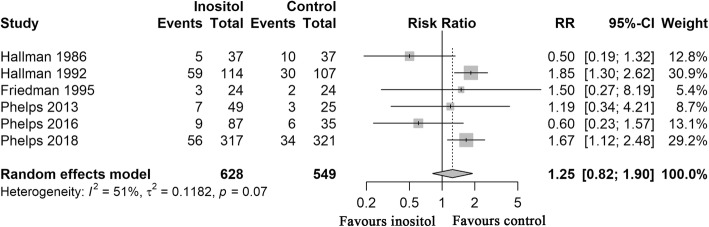
Forest Plot Showing Risk Ratio (RR) in Mortality**.** RR indicates risk ratio; CI, confidence interval. Risk ratios were calculated using the DerSimonian-Laird method to combine summary statistics, and data were pooled using a random-effects model

Mortality was reported in six RCTs [15, 16, 19, 24–26], overall mortality was 139 (22.13%) of the 628 preterm infants in the inositol group versus 85 (15.48%) of the 549 preterm infants. The results of meta-analysis revealed no statistically significant difference between infants who received inositol supplement versus placebo (RR = 1.25, D-L random-effects, 95% CI, 0.82–1.90; heterogeneity, *P* = .07; I^2^ = 51%; low QOE; Fig. 3**,** Table 3).

### Secondary outcomes

#### Any stage of ROP

**Fig. 4:**
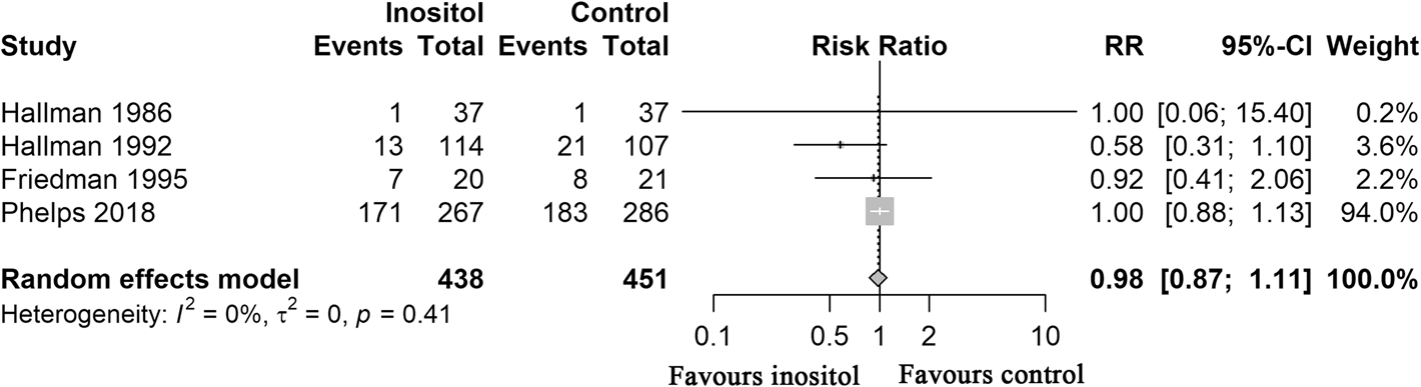
Forest Plot Showing Risk Ratio (RR) in Any Stage of ROP. RR indicates risk ratio; CI, confidence interval; ROP, retinopathy of prematurity. Risk ratios were calculated using the DerSimonian-Laird method to combine summary statistics, and data were pooled using a random-effects model

Any stage of ROP was reported in four studies [15, 16, 19, 23]. The result showed that 192 of 438 (43.84%) preterm infants developed ROP in the inositol group versus 213 (47.23%) of 451 in the placebo group. The meta-analysis revealed no statistically significant difference among infants who received inositol supplement versus those who received a placebo (RR = 0.98, D-L random-effects, 95% CI, 0.87–1.11; heterogeneity, *P* = .41; I^2^ = 0%; moderate QOE; Fig. 4**,** Table 3).

### Adverse events

No statistically significant difference was detected between the inositol and control group on the incidence of BPD (RR = 0.95, D-L random-effects, 95% CI, 0.72–1.25; heterogeneity, *P* = .15; I^2^ = 38%; low QOE), suspected or proven NEC (RR = 0.85, D-L random-effects, 95% CI, 0.58–1.24; heterogeneity, *P* = .48; I^2^ = 0%; moderate QOE), surgical NEC (RR = 0.76, D-L random-effects, 95% CI, 0.24–2.38; heterogeneity, *P* = .15; I^2^ = 48%; moderate QOE), all stage of IVH (RR = 0.77, D-L random-effects, 95% CI, 0.59–1.00; heterogeneity, *P* = .45; I^2^ = 0%; moderate QOE), severe IVH (grade III/IV) (RR = 0.68, D-L random-effects, 95% CI, 0.45–1.03; heterogeneity, *P* = .17; I^2^ = 35%; low QOE), late-onset sepsis (RR = 1.22, D-L random-effects, 95% CI, 0.97–1.54; heterogeneity, *P* = .40; I^2^ = 0%; low QOE), seizure (RR = 1.02, D-L random-effects, 95% CI, 0.41–2.56; heterogeneity, *P* = .34; I^2^ = 8%; high QOE), hearing impairment (RR = 1.36, D-L random-effects, 95% CI, 0.87–2.31; heterogeneity, *P* = .48; I^2^ = 0%; high QOE) (See Additional file 1: Figure S1).

#### Sensitivity analysis and Meta-regression

We estimated the pooled effect of the primary outcomes of inositol supplement compared with placebo after removing the study Hallman 1986 [15], which was the only study not using surfactant. Without the study, the results of meta-analysis showed an increased mortality in the inositol group, with a risk ratio of 1.55 (D-L random-effects, 95% CI, 1.14–2.11; heterogeneity, *P* = .30; I^2^ = 18%).

**Fig. 5:**
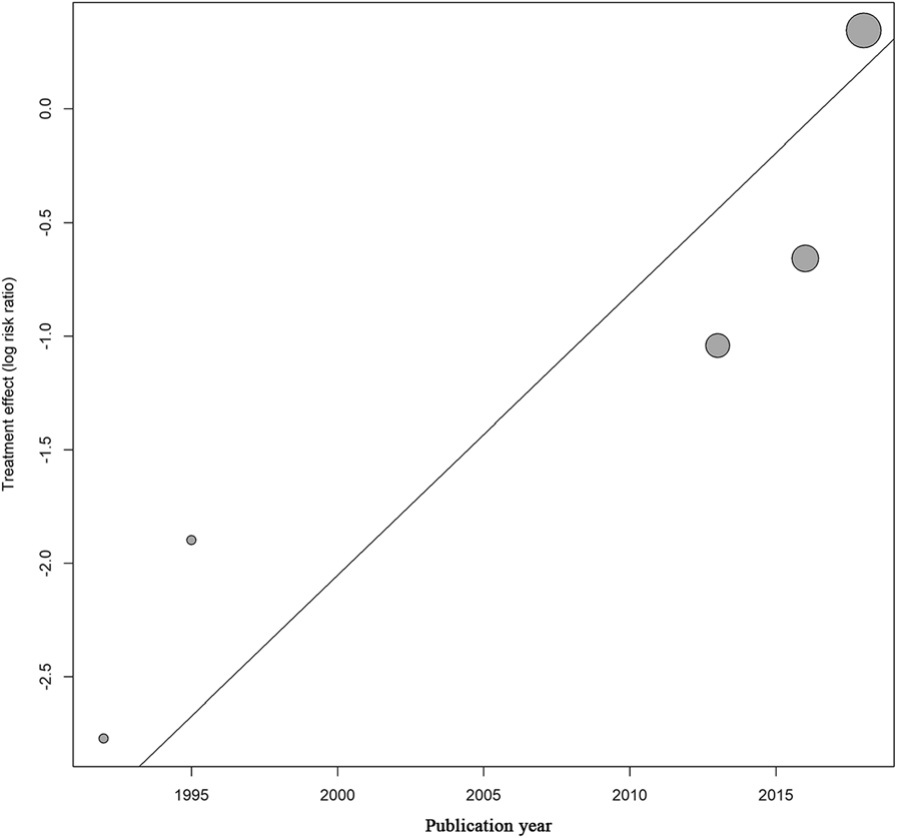
Bubble Diagram Examining Relationship Between Publication Year and Efficacy of Inositol Compared with Placebo. RR indicates risk ratio

We additionally examined the relationship between publication year and primary outcomes. Meta-regression demonstrated a significant association between publication year and measured efficacy of inositol compared with placebo (β = 0.1241; 95% CI, 0.0417–0.0026; z = 2.9527; *p* = .0032) (Fig. 5). However, there was no significant association between publication year and risk ratio of mortality in inositol group compared with placebo group (β = 0.0045; 95% CI, − 0.0382–0.0473; z = 0.2081; *p* = .8351) (See Additional file 1: Figure S2).

## Discussion

To our knowledge, this is the most up-to-date meta-analysis to determine the efficacy and safety of inositol supplementation in preterm infants for preventing ROP, and our result has challenged conclusions from previous systematic reviews [17, 18]. In this meta-analysis, we synthesized the evidence from six RCTs including 1194 preterm infants to describe the effect of inositol administration in preterm infants. On the basis of low quality of evidence, we found that inositol has no effect on the incidence of severe ROP and mortality. Inositol has no effect on the incidence of all stage of ROP, BPD, suspected or proven NEC, surgical NEC, all grade of IVH, severe IVH (grade III or IV), neonatal seizures and hearing impairment, either.

Some studies have demonstrated some important biological functions of inositol, including the adjustment of cell osmotic pressure [27], the maturity of nervous system [28], and the synthesis of pulmonary surfactant phospholipid [29], etc. Evidence has shown that inositol was rich in the umbilical artery of embryo [30], suggesting inositol might be one of the basic substances for human growth. Meanwhile, the concentration of inositol was high in human breast milk (> 1200 mmol/L), which means inositol was an important need for infants after birth [31].

However, the results of our meta-analysis didn’t support a positive effect of inositol in preventing severe ROP in preterm infants. Our results even concluded that inositol may have a potential trend to increase the mortality of infants, and the heterogeneity among included studies was high. In the early studies [15, 16, 23, 25, 26], the results favored the benefits of inositol to prevent severe ROP and other preterm comorbidities, but the beneficial findings were not observed in the last study reported by Phelps 2018 [19]. The meta-analysis and sensitivity analysis might give us some explanations about the high heterogeneity. For the efficacy of inositol decreasing along with the publication year, one explanation could be that inositol may promote surfactant synthesis and function. In the early trials, antenatal steroids and surfactant were not widely used, inositol could reduce the severity of respiratory distress syndrome, and by the way, reduce ROP and other morbidities. However, antenatal steroids, exogenous surfactant, and noninvasive ventilator support have been more and more widely used during the past 30 years [32–34], the use of antenatal steroids and exogenous surfactant have outweighed the benefit of inositol. And the definition severe ROP which needs intervention has changed in the more than 30 years due to the advanced treatment such as laser treatment and anti-VEGF agents. Besides, Phelps 2018 [19] reported a significant increase in mortality has challenged the safety of inositol, opposite to the results of other five small-size studies [15, 16, 23, 25, 26] without enough power. Although the result of meta-analysis revealed no statistically significant difference of the mortality between the two groups, there was a trend toward an increase on mortality of infants in the inositol group. The sensitivity analysis after removing the study Hallman 1986 [15], which was the only study not using surfactant, showed a statistically significant increased mortality in the inositol group. One explanation could be that side effects appeared after the benefit of inositol was outweighed by surfactant. Additionally, the difference of participants, inositol provided for the trials, dose of inositol and the duration of treating might also play a role in the results. For example, the infants included in Phelps 2018 [19] were more immature than the prior studies, and the duration of treating in this study was up to 10 weeks, much longer than the prior studies.

### Strengths and limitations

The strengths of this review include explicit eligibility criteria; a comprehensive search peer-reviewed by a research librarian, with no language restriction; independent assessment of eligibility, data abstraction, assessment of risk of bias, quality of evidence assessment by using the GRADE approach; registration on the PROSPERO website and report followed the QUOROM statement [35], the guidelines for meta-analysis of RCTs. Our meta-analysis updated the Cochrane review published in 2015 [17] and included other two RCTs [19, 26] published recently. We also drew a conclusion which is totally different from the previous meta-analysis, which reminded inositol administration cautiously.

Our meta-analysis also had several limitations. Firstly, only three RCTs were rated as high quality and the other three were varying from moderate to low, which along with the high heterogeneity led to low QOE of primary outcomes. Secondly, we did meta-regression to examine the relationship between publication year and primary outcomes, which had relatively low power when few studies were included in the analysis. Thirdly, we couldn’t evaluate publication bias statistically via funnel plot due to the number of trials included were less than ten. Besides, we lacked individual patient data to find out the potential beneficial subgroup. Finally, all the included studies lacked long-term follow-up outcomes, such as visual outcomes and structural outcomes in childhood.

## Conclusions

The present meta-analysis showed that inositol supplementation may have no effect in prevention of severe ROP but a trend toward an increase on mortality in preterm infants less than 32 weeks. Routine inositol supplementation to preterm infants should not be recommended based on current evidence.

## Additional file

Additional file 1: **Figure S1.** Forest Plot Showing Risk Ratio (RR) in Complications and Adverse Event. **Figure S2.** Bubble Diagram examining Relationship Between Publication Year and Mortality. (DOCX 595 kb)

## Data Availability

All data generated or analysed during this study are included in this published article [and its supplementary information files].

## Abbreviations

CI: Confidence interval
QOE: Quality of evidence
RCT: Randomized controlled trial
ROP: Retinopathy of prematurity
RR: Relative risk
