# Supplementary material for: The efficacy and safety of inositol supplementation in preterm infants to prevent retinopathy of prematurity: a systematic review and meta-analysis
Source: BMC Ophthalmol. 2019 Jun 25;19:135. doi: 10.1186/s12886-019-1140-z (PMC6593579; doi:10.1186/s12886-019-1140-z)
Supplement: Supplementary file 1 — Figure S1. Forest Plot Showing Risk Ratio (RR) in Complications and Adverse Event. Figure S2. Bubble Diagram examining Relationship Between Publication Year and Mortality. (DOCX 595 kb) [file 12886_2019_1140_MOESM1_ESM.docx]

**Additional Files**

Figure S1. Forest Plot Showing Risk Ratio (RR) in Other Adverse Events

Figure S2. Bubble Diagram examining Relationship Between Publication Year and Mortality

**Figure S1. Forest Plot Showing Risk Ratio (RR) in Complications and Adverse Event**


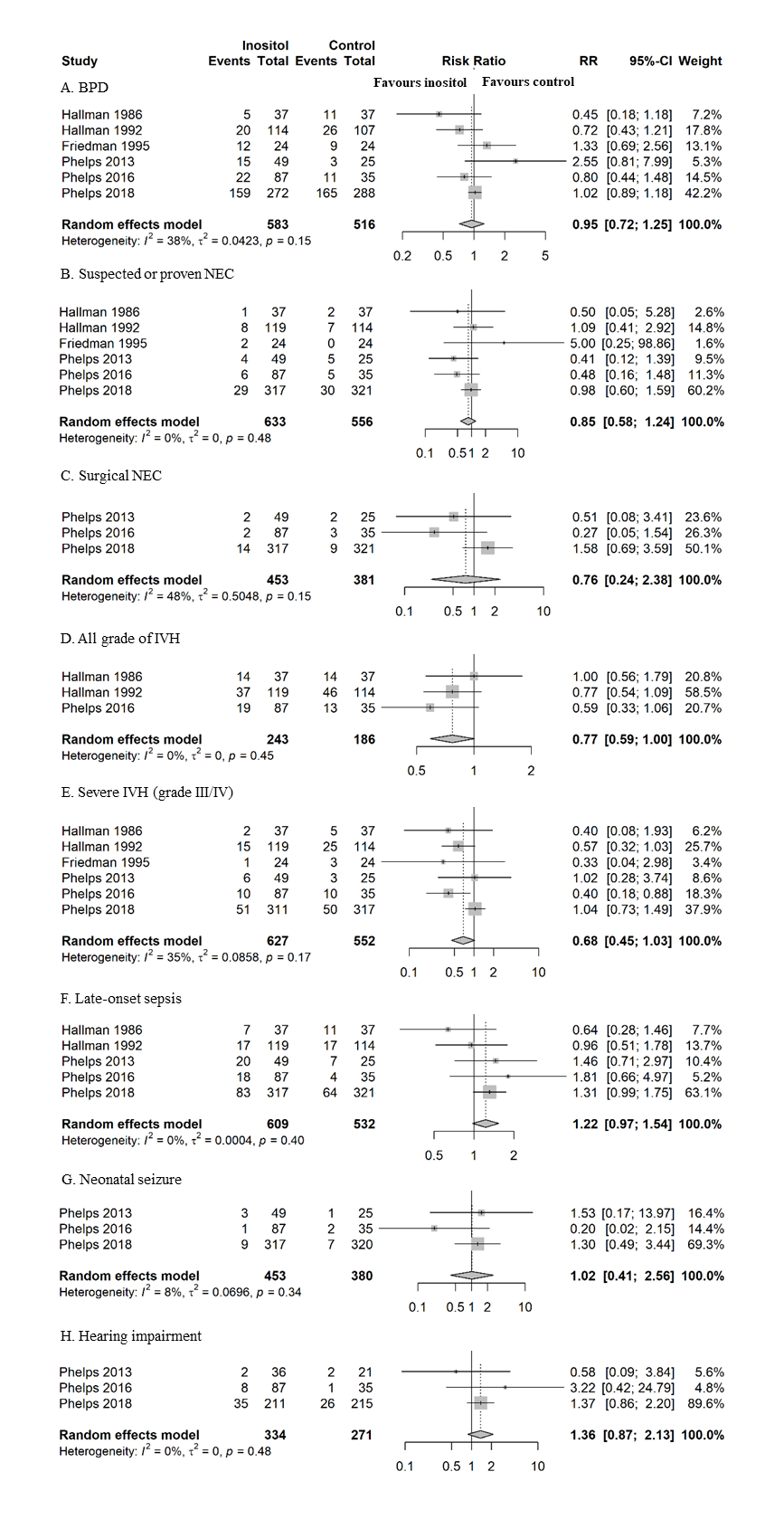


RR indicates risk ratio; CI, confidence interval; BPD, Bronchopulmonary dysplasia; NEC, necrotizing enterocolitis; IVH, intraventricular hemorrhage.

**Figure S2. Bubble Diagram examining Relationship Between Publication Year and Mortality**


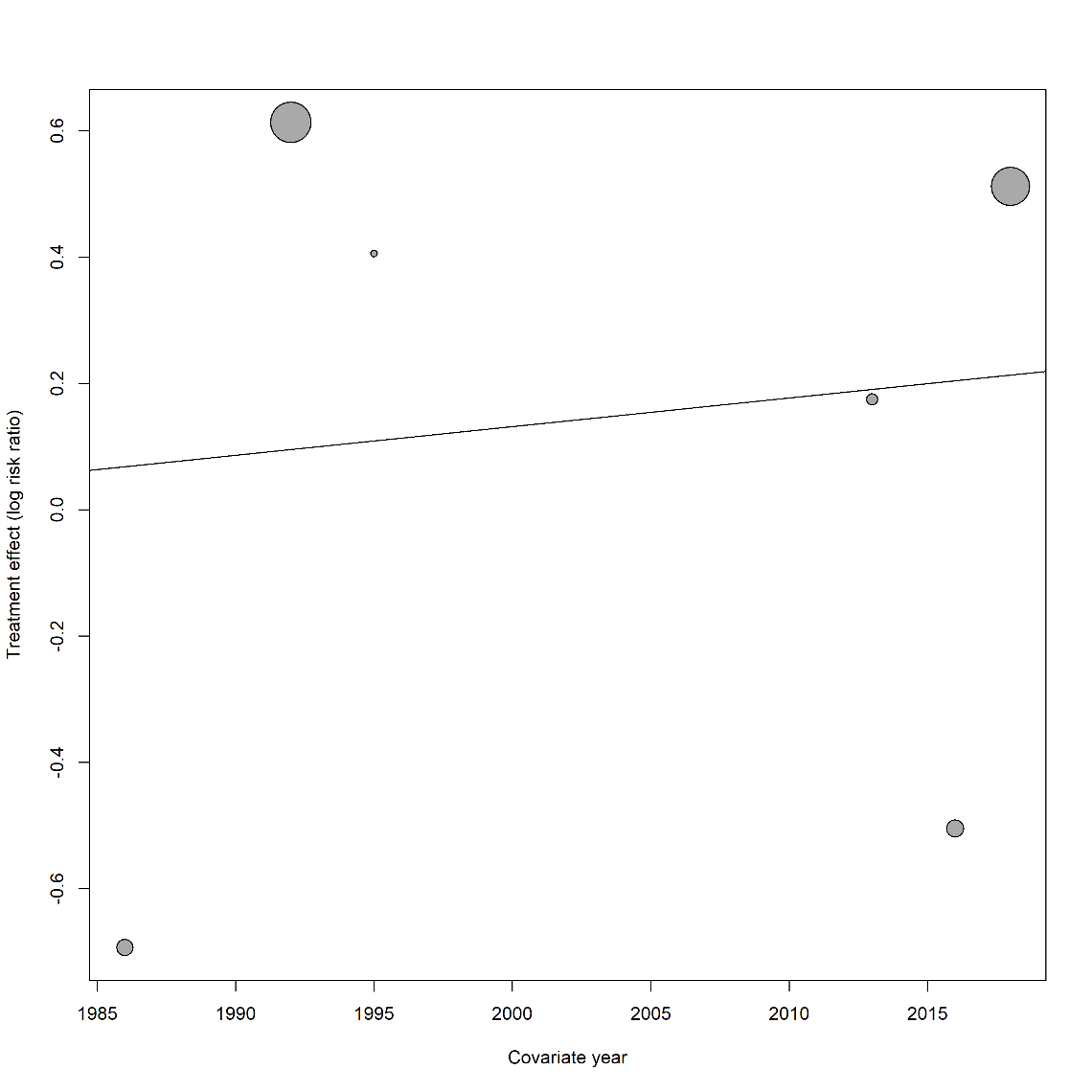


Publication year

RR indicates risk ratio.
